# Genetic Polymorphisms in ADORA2A and CYP1A2 Influence Caffeine’s Effect on Postprandial Glycaemia

**DOI:** 10.1038/s41598-019-46931-0

**Published:** 2019-07-19

**Authors:** N. F. Banks, P. M. Tomko, R. J. Colquhoun, T. W. D. Muddle, S. R. Emerson, N. D. M. Jenkins

**Affiliations:** 1 Applied Neuromuscular Physiology Laboratory, Oklahoma State University, Stillwater, OK USA; 2 Laboratory for Applied Nutrition and Exercise Science, Oklahoma State University, Stillwater, OK USA; 3 Nutritional Sciences, Oklahoma State University, Stillwater, OK USA

**Keywords:** Metabolism, Predictive markers

## Abstract

The liver enzyme cytochrome P450 1A2 (CYP1A2) is responsible for 90% of caffeine metabolism, while caffeine exerts many of its effects via antagonist binding to adenosine A2a receptors (ADORA2A). This study aimed to examine whether functional single nucleotide polymorphisms (SNPs) in 1976T > C (ADORA2A; rs5751876) and −163C > A (CYP1A2; rs762551) influence the effect of caffeine on the postprandial glucose (GLU) response to a carbohydrate meal. We report that individuals with the 1976T > C CC, but not CT/TT genotypes display elevated GLU levels after consuming caffeine and carbohydrate (CHO + CAFF) versus carbohydrate only (CHO). The GLU area under the curve (AUC) was also greater during the CHO + CAFF condition compared to the CHO condition in CC, but not the CT/TT genotypes. The −163C > A AC/CC, but not AA, genotypes displayed greater GLU concentrations 60-min post meal during CHO + CAFF versus CHO. Our data suggest that caffeine-induced impairments in postprandial glycaemia are related to 1976T > C and −163C > A SNPs.

## Introduction

Caffeine is the most widely consumed psychostimulant in the world and^1^, when consumed, affects virtually every tissue in the body^2^. In the US, children and adolescents ingest the majority of their caffeine from carbohydrate-containing beverages, which are also second only to coffee in adults^3^. It is also not uncommon for adults to consume coffee or other caffeine-containing beverages at meals containing high carbohydrate content^4^. In the past, oral glucose tolerance tests (OGTT) have been used to examine the postprandial response to a carbohydrate and caffeine load, and studies observing glucose (GLU) or insulin activity following acute caffeine consumption show an overall negative effect^5–9^. For example, Lane *et al*. observed GLU and insulin levels for 2 hours following a mixed-meal tolerance test and found that when subjects consumed 375 mg of caffeine prior to meal consumption, GLU and insulin AUC increased 21% and 48% respectively^10^. Similarly, Graham *et al*. observed that a 5 mg/kg caffeine load ingested prior to a OGTT resulted in a 60% larger AUC for insulin in the 2 hours post consumption^7^. Interestingly, epidemiological studies have suggested that habitual coffee consumption may be associated with decreased risk for Type 2 diabetes^11^. Nevertheless, coffee contains chlorogenic acid, quinic acid, trigonelline, and lignin secoisolariciresinol, which all have been shown to improve glucose metabolism^12^. This is supported by data indicating that caffeine alone significantly elevated glucose, insulin, and C-peptide levels following an OGTT compared to decaffeinated coffee. Caffeinated coffee blunted these negative effects exhibited by caffeine, but still resulted in a trend towards an elevated glucose response compared to placebo and decaffeinated coffee. Further, decaffeinated coffee resulted in a 50% decrease in glucose area under the curve (AUC) compared to placebo^13^. Therefore, the effects of coffee versus caffeine may not be the same, and the positive health benefits of coffee on Type 2 diabetes are likely not attributed to its caffeine content. Thus, clearly, studies are needed to further understand the influence of caffeine on postprandial glycemic responses.

Caffeine and its metabolites theophylline and paraxanthine are potent adenosine receptor antagonists^14,15^, and the physiological effect of caffeine has been largely attributed to this competitive binding, specifically to the A2a receptor (ADORA2A)^16^. Adenosine is the product of adenosine 5-triphosphate (ATP) metabolism, and has been shown to inhibit the sympathetic nervous system and, in particular, norepinephrine (NE) and epinephrine (E) release^17^, which are responsible for inhibiting glucose disposal and upregulating glucose mobilization, respectively^18^. In accordance, methylxanthines have been shown to drastically increase NE and E levels due to the antagonization of adenosine receptors and their excitatory influence on the sympathetic nervous system^19^ is a possible mechanism behind previous reports of caffeine-induced increases in postprandial glycemia^5,6,9,20^. A functional, single nucleotide polymorphism (SNP) in ADORA2A (1976T>C; rs5751876), formally known as 1083 T > C, has been identified that appears to influence physiological and psychological responses to caffeine. For example, Retey *et al*.^18^ reported significantly higher spectral power in the beta band of electroencephalogram (EEG) after consuming 200 mg caffeine in the CC but not TT genotype. These results were further replicated by observing the SNPs rs5760423, rs5760425, and rs3761422, which are in perfect linkage disequilibrium with rs5751876^21^. Both Alsene *et al*.^22^ and Childs *et al*.^23^ reported that subjects with the TT genotype experienced greater self-perceived anxiety following caffeine consumption than the CC genotype. Subsequently, the ADORA2A1976T > C substitution may be of interest when examining individual variability in glycemic responses to a caffeinated carbohydrate load.

Quantifying the body’s ability to metabolize caffeine is often accomplished through examination of the ratio of paraxanthine to caffeine (17X/137X) after caffeine consumption^24^. The CYP1A2 enzyme is responsible for 90% of caffeine metabolism, and for 84%, 12%, and 4% of the metabolism of caffeine’s metabolites: paraxanthine, theophylline, and theobromine, respectively^16^. A SNP in the CYP1A2 enzyme (−163C > A; rs762551) has been suggested to influence the inducibility of CYP1A2 activity. Specifically, the −163C > A substitution has been shown to alter the rate of caffeine metabolism in smokers^25^ and heavy coffee consumers^26^. Consequently, −163C > A genotype has been differentially associated with caffeine’s effect on exercise performance^27,28^, risk of myocardial infarction^29^, sleep^30^, and blood pressure^31^. To our knowledge, only Robertson *et al*.^8^ has examined how −163C > A genotype influences metabolic responses in a post-prandial period. Using a liquid mixed-meal tolerance test after chronic coffee consumption, they reported that AA genotypes exhibited higher postprandial GLU levels but lower free fatty acid concentrations compared to CC genotypes. Insight into alterations in postprandial metabolism based on −163C > A genotype may be useful for individualization of nutrition programs in the future.

The purpose of this study was to examine if 1976T > C (ADORA2A; rs5751876) and −163C > A (CYP1A2; rs762551) SNPs influence the effect of caffeine on the GLU response to a carbohydrate meal. Based on previous reports indicating differences in psychological and physiological responses to caffeine^18^, we hypothesized that there would be differences in the GLU response after caffeine consumption in the ADORA2A CC versus CT/TT genotype. In addition, based on evidence that the −163C > A SNP has only been shown to exhibit an effect on CYP1A2 enzyme inducibility in smokers^25^ and heavy caffeine consumers^26^, it was hypothesized that there would be no difference in GLU levels between CYP1A2 AA versus AC/CC genotypes.

## Methods

### Experimental design

**Figure 1:**
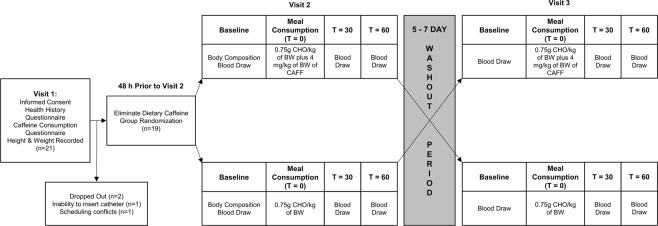
A detailed overview of the study design. BW = Body Weight; CAFF = Caffeine; CHO = Carbohydrate; T = Time.

A randomized, single-blind, cross-over design was used for this study, which consisted of three visits. During visit 1, the participants completed a validated caffeine consumption questionnaire^32^ and had their height and weight measured. Visit 2 and 3 were conducted at the same time of day (±15 min), with 5–7 days between visits. Prior to visits 2 and 3, participants were asked to abstain from caffeine, log food consumption for the preceding 48 h, and abstain from exercise for at least the preceding 36 h. Participants arrived at the laboratory following a 10 hour fast for visits 2 and 3, completed the International Physical Activity Questionnaire (IPAQ), and completed body composition assessments. A baseline blood draw was then performed. Following the baseline assessments, participants consumed either a liquid CHO meal or the same liquid CHO meal with pharmaceutical grade caffeine added (CHO + CAFF). Blood draws were again performed 30- and 60-min post-meal. Figure 1 provides a detailed overview of the study design.

### Participants

Twenty healthy males volunteered to participate in this study, however only eighteen (mean ± SD, age = 24.84 ± 3.61 y, weight = 93.33 ± 15.79 kg, height = 176.66 ± 6.55 cm, %BF = 17.63 ± 7.35) completed the full protocol. Two participants did not finish the study due to difficulties with catheter insertion (n = 1) or scheduling conflicts (n = 1). In order to be eligible for this study, each participant must have been a male between the ages of 18 and 35 years, free of any cardiovascular, metabolic, or neuromuscular disease, a non-smoker, and could not have been currently taking any medications known to alter caffeine or energy metabolism. Prior to any data collection, all participants completed an informed consent form, a health history questionnaire, and a habitual caffeine consumption questionnaire^32^. For this initial study, only men were recruited because caffeine metabolism can be affected by oral contraceptives and is slowed during the luteal phase of the menstrual cycle in women^33^. Participants reported consuming an average of 124.9 ± 137.5 mg of caffeine per day, ranging from 3–394.26 mg per day. The participants were genotyped for rs5751876 (CC; n = 7, CT/TT; n = 11) and rs762551 (AA; n = 11, AC/CC; n = 7) while participating in a previous study^34^. The observed genotype frequencies for 1976T > C^23,35^ and −163C > A^24,25^ were similar to those reported previously. This study was approved by and conducted in accordance to the guidelines and regulations of the Oklahoma State University’s Institutional Review Board (IRB; Approval #: ED-18–87, Approval Date: July 19, 2018).

### Sample size determination

We utilized a three-pronged approach to determine our sample size, as follows. First, we estimated the sample size required to observe a within-between interaction using a two-way mixed factorial ANOVA using medium effect size of 0.25 and standard power of 0.8 with a conservative correlation between measurements of 0.8. This analysis resulted in an estimated total sample size of 12 to detect an interaction effect if one were present. Second, we estimated the sample size needed to observe a within-group difference in the CAFF + CHO versus CHO condition at 60 minutes in the individuals with the −163C > A AA genotype, which was conducted using an estimated effect size of 1.01, per Robertson *et al*.^8^, and a power of 0.8. This analysis resulted in a required sample size of 8 subjects per group. Finally, a total sample size of 19 was used by Robertson *et al*. (2018) and was sufficient to detect differences in post-prandial glucose responses among −163C > A genotypes following chronic caffeine administration. Therefore, we recruited and enrolled 20 participants in this study.

### Genotyping

Genotyping was performed as described in detail previously^36^. In brief, an Oragene ON-500 saliva collection kit (DNA Genotek, Ottawa, Ontario, Canada) was used to collect saliva samples from each participant for DNA analysis. The DNA samples were then shipped to the University of Toronto where they were stored at −80 °C until analysis. Genotyping of the rs762551 (−163C > A) SNP and of the rs5751876 (1976T > C) SNP were performed using the iPLEX Gold assay with mass-spectrometry-based detection on the Sequenom MassARRAY® platform (Agena Bioscience, San Diego, CA, USA) as described previously^37^.

### Liquid meal

The CHO + CAFF and CHO meal were administered as a liquid supplement, which was prepared the morning of each visit. The CHO meal served as the placebo, and consisted of 0.75 g/kg of CHO in the form of sucrose and dextrose (Gatorade® Fruit Punch Thirst Quencher Powder, Chicago, IL, USA) mixed with 7.5 ml/kg of water. The CHO + CAFF meal consisted of the same dose of CHO and water, except that 4 mg/kg of pharmaceutical grade caffeine anhydrous powder (98.5–101.0% USP, CSPC Innovation Pharmaceutical Co., LTD) was also mixed in the drink. At each visit, the average meal consumed was 70.67 ± 11.59 g CHO mixed with 706.73 ± 115.88 ml of water and, during the CHO + CAFF condition only, 376.92 ± 61.81 mg CAFF (range = 226.2–479.6 mg). The liquid meal was consumed after baseline measurements and the participants were instructed to drink the meal quickly.

### Metabolic assessment

A 24-guage indwelling safelet catheter (Exelint International, Redondo Beach, CA, USA) was inserted into a forearm vein and kept clear with a consistent infusion of 0.9% NaCl solution (~1 drip per second), while a tegaderm film (3M Healthcare, Neuss, Germany) was applied in order to keep the catheter stationary. After catheter set up, a blood draw was taken at baseline, followed by subsequent draws at 30- and 60-min post meal consumption. During each blood draw, a 3 mL syringe was used to clear saline from the line before a sample of whole blood was collected using a 5 mL syringe (BD, Franklin Lakes, NJ, USA). A few drops of whole blood from each blood draw were transferred into a Cholestech LDX (Alere Cholestech, San Diego, CA, USA) via a capillary tube and analyzed to determine glucose concentrations ([GLU], mg/dL; coefficient of variation = 3–5%). Participants stayed seated in the laboratory during the hour following the consumption of the liquid meal.

### Blood pressure

Blood pressure was assessed by following the American Society of Hypertension guidelines^38^ using an automatic blood pressure cuff (Omron 3 Series Upper Arm, Kyoto, Japan) during visit 2.

### Body composition

During visit 1, participant’s height (Physician Scale 439; Detecto, Webb City, MO, USA) and weight (ADAM CPWplus 150 M, Oxford, CT, USA) were collected. On visit 2, body mass index (BMI), fat free mass (FFM), fat mass (FM) and fat mass percent (%FM) was assessed via bioelectrical impedance spectroscopy (BIS) (ImpediMed SFB7, Carlsbad, CA, USA) in accordance to the manufacturer recommended procedures as described in detail previously^34,39^.

### Lifestyle controls

Participants were instructed to keep their dietary and physical activity habits consistent throughout the course of the study. Upon entering the lab for visits 2 and 3, participants turned in a dietary log in which they had recorded all food and calorie-containing beverages consumed during the two days prior to each visit. Participants were instructed to record all dietary information immediately after consumption, while providing information regarding the method of preparation and serving size. This information was then entered into the ESHA’s Food Processor® nutrition analysis Software (https://www.esha.com, ESHA Research, Oak Brook, Il, USA) that provided calculations of absolute daily energy (kcals), and fat (g), carbohydrate (g), and protein (g) intakes^40,41^. The average daily kcals, protein, carbohydrate, and fat consumed prior to visits 2 and 3 were then used for analyses. In order to assess physical activity levels during the days leading up to visits 2 and 3, participants filled out the International Physical Activity Questionnaire Long Last 7-days Format (IPAQ)^42^. The IPAQ provided metabolic equivalent for tasks (MET) minutes performed during the week prior to visits 2 and 3.

### Statistical analyses

Independent samples t-tests were used to ensure no differences in height, weight, %FM, and BMI between CYP1A2 AA vs. AC/CC or ADORA2A CC vs. CT/TT genotypes. Separate two-way (genotype [CYP1A2 AA vs. AC/CC or ADORA2A CC vs. CT/TT] × condition [CHO vs. CHO + CAFF]) mixed factorial ANOVAs were used to ensure no differences in MET·min·week^-1^, or in total kcal, fat (g), carbohydrate (g), and protein (g) intake among genotypes prior to the CHO and CHO + CAFF visits. Two-separate, three-way (genotype [CYP1A2 AA vs. AC/CC or ADORA2A CC vs. CT/TT] × condition [CHO vs. CHO + CAFF] × time [BL vs. 30-min vs. 60- min]) mixed factorial ANOVAs were used to analyze GLU responses. In addition, trapezoidal areas under the GLU curve (AUCs) were calculated for each participant during the CHO and CHO + CAFF conditions using Excel 2016. Subsequently, GLU AUCs were analyzed with two- separate, two-way mixed factorial ANOVAs (genotype [CYP1A2 AA vs. AC/CC or ADORA2A CC vs. CT/TT] × condition [CHO vs. CHO + CAFF]). When appropriate, follow-up analyses of all ANOVAs included lower-order ANOVAs and Sidak multiple comparison tests. All statistical analyses were performed using GraphPad (GraphPad Software v. 8, La Jolla, CA, USA) and SPSS (v. 24; International Business Machines Corp., Armonk, NY, USA). The type-I error rate was set a priori at 5%.

## Results

### Body composition

No significant differences in weight, FFM, FM, %FM, or BMI were found among the CYP1A2 AA vs. AC/CC or ADORA2A CC vs. CT/TT genotype (Table 1). There was no significant difference in height between ADORA2A CC vs. CT/TT genotype, but height was greater in the CYP1A2 AC/CC than AA group (Table 1).

**Table 1:** Mean ± SD height, weight, fat free mass, fat mass, and %FM of the −163AA vs. AC/CC and 1976CC vs. CT/TT genotype.

	−163C > A (n = 18)			1976T > C (n = 18)		
	AA (n = 11)	AC/CC (n = 7)	p-value	CC (n = 7)	CT/TT (n = 11)	p-value
Height (cm)	174.5 ± 5.6	181.1 ± 6.2	p = 0.031*	177.2 ± 6.7	176.9 ± 6.8	p = 0.947
Weight (kg)	94.3 ± 18.6	94.2 ± 11.7	p = 0.995	86.6 ± 17.3	99.1 ± 13.5	p = 0.104
Fat Free Mass (kg)	77.6 ± 13.2	75.7 ± 13.2	p = 0.747	82.0 ± 13.8	73.6 ± 10.0	p = 0.155
Fat mass (kg)	17.3 ± 8.4	17.5 ± 9.9	p = 0.995	18.8 ± 9.1	16.4 ± 8.8	p = 0.589
Fat Mass Percent (%)	17.4 ± 7.3	18.8 ± 8.2	p = 0.723	16.5 ± 8.2	18.9 ± 7.2	p = 0.529
Body Mass Index	30.9 ± 2.7	28.7 ± 5.8	p = 0.354	27.4 ± 4.2	31.7 ± 4.6	p = 0.065

### Physical activity

There was no significant genotype × condition interaction for MET·min/week when examining CYP1A2 − 163C > A or ADORA2A 1976T > C genotypes, nor a main effect for condition or genotype (Table 2).

**Table 2:** Dietary intake and physical activity levels averaged over the two days and week prior to each visit respectively.

	−163C > A (n = 18)		1976T > C (n = 18)		Combined (n = 18)	P-value		Condition
	AA (n = 11)	AC/CC (n = 7)	CC (n = 7)	CT/TT (n = 11)		Condition x −163C > A	Condition x 1976T > C	
**Calories (kcal)**								
CHO	2291.49 ± 526.94	2123.73 ± 792.69	2403.06 ± 551.30	2113.76 ± 669.75	2226.25 ± 626.25	p = 0.408	p = 0.850	p = 0.872
CHO + CAFF	2376.99 ± 594.74	1997.43 ± 403.50	2376.31 ± 417.44	2135.89 ± 619.48	2229.38 ± 549.34			
**Fat (g)**								
CHO	90.38 ± 31.23	92.56 ± 33.84	83.90 ± 18.42	95.89 ± 37.40	91.23 ± 31.29	p = 0.476	p = 0.228	p = 0.550
CHO + CAFF	95.28 ± 32.39	89.43 ± 36.11	93.84 ± 27.34	92.47 ± 33.00	93.01 ± 32.95			
**Carbohydrates (g)**								
CHO	231.19 ± 79.38	218.84 ± 149.83	262.68 ± 111.21	203.30 ± 104.43	226.39 ± 140.55	p = 0.495	p = 0.963	p = 0.826
CHO + CAFF	241.16 ± 69.13	199.445 ± 84.05	260.01 ± 55.00	202.62 ± 80.86	224.94 ± 75.78			
**Protein (g)**								
CHO	124.37 ± 41.39	134.08 ± 48.89	165.96 ± 28.82	124.37 ± 41.39	140.55 ± 41.67	p = 0.974	p = 0.410	p = 0.223
CHO + CAFF	134.69 ± 53.48	124.74 ± 42.55	146.92 ± 57.16	120.58 ± 41.61	130.82 ± 48.44			
**MET·min·week** ^**−1**^								
CHO	3915.63 ± 1252.76	4737.57 ± 1659.86	4503.93 ± 1660.48	4064.31 ± 1332.10	4235.27 ± 1437.21	p = 0.217	p = 0.471	p = 0.518
CHO + CAFF	3763.22 ± 1407.92	4324.10 ± 2136.94	4393.21 ± 1995.88	3719.24 ± 1507.41	3981.34 ± 1690.23			

### Dietary intake

There was no significant genotype × condition interaction for daily average kcal, fat, carbohydrate, or protein consumption when examining CYP1A2 −163C > A or ADORA2A 1976T > C genotypes, nor a condition or genotype main effect (Table 2).

### Blood glucose

#### ADORA2A (1976T > C)

**Figure 2:**
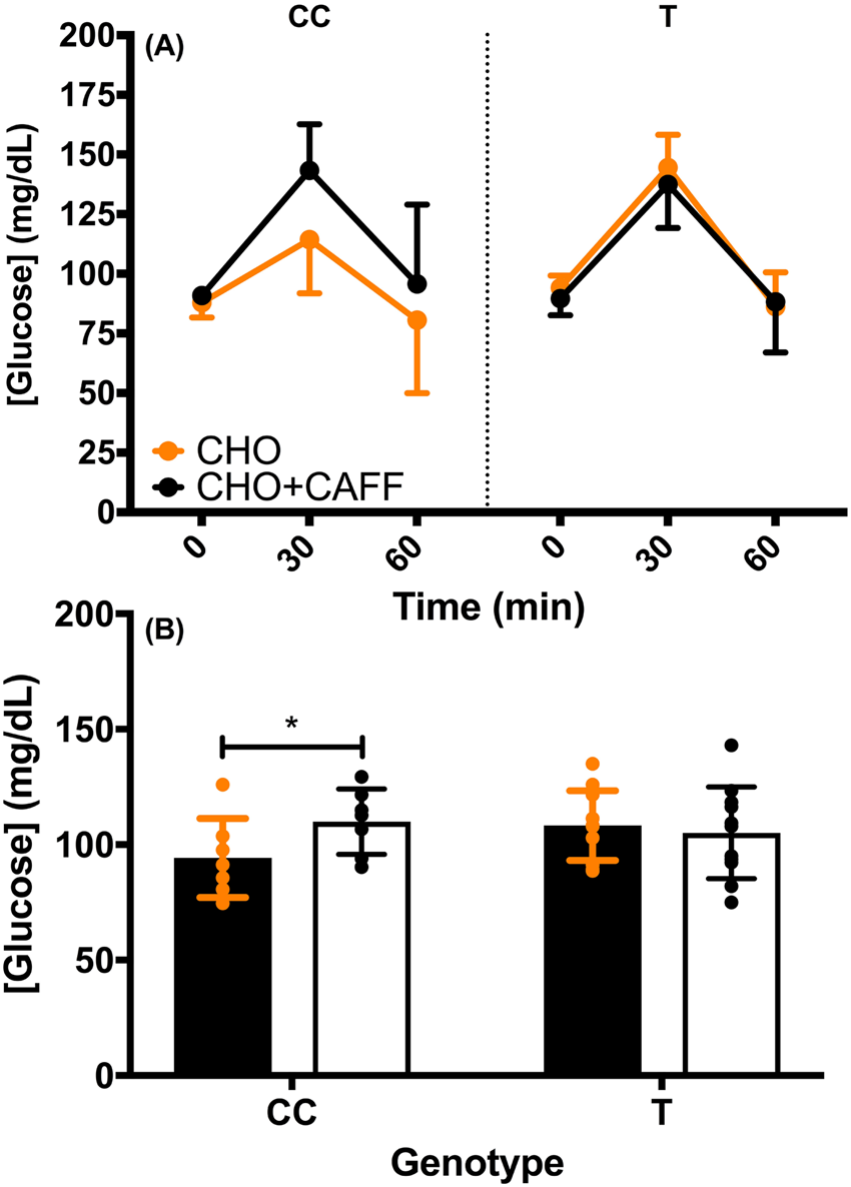
(**A**) The mean ± 95% CI glucose concentrations for the ADORA2A 1976CC versus CT/TT (denoted as T) genotypes during the carbohydrate only (CHO; orange data points) versus carbohydrate and caffeine (CHO + CAFF; black data points) conditions. **(B)** The mean (collapsed across time) ± 95 CI glucose concentrations for the 1976CC versus CT/TT genotypes during the carbohydrate only (closed black bars, orange circles) and carbohydrate and caffeine (open black bars, black circles) conditions. * indicates a significant difference between conditions for the CC genotype, p < 0.05.

**Figure 3:**
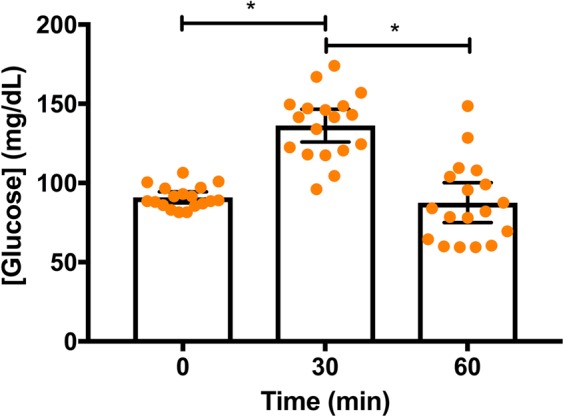
The mean (collapsed across condition and genotype) ± 95% confidence interval glucose concentrations at BL, and 30- and 60-min post meal consumption. * indicates a significant difference between time points, p < 0.05.

There was no significant genotype × condition × time interaction (F_2,15_ = 3.042, p = 0.078, np^2^ = 0.289) observed for [GLU] when examining ADORA2A 1976T > C genotype (Fig. 2A). There was, however, a significant genotype × condition interaction (F_1,16_ = 7.24, p = 0.02, np^2^ = 0.31; Fig. 2). [GLU] was significantly greater during the CHO + CAFF than CHO condition in the CC group (mean ± 95% CI difference = 15.71 ± 11.71 mg/dL), whereas [GLU] was the same during the CHO and CHO + CAFF conditions in the CT/TT genotype (mean ± 95% CI difference = 3.15 ± 10.46 mg/dL; Fig. 2B). There was also a significant main effect for time (F_2,15_ = 81.456, p < 0.001, np^2^ = 0.916; Fig. 3). [GLU] increased from baseline to 30-min (mean ± 95% CI difference = 45.36 ± 8.26 mg/dL), before decreasing back to baseline concentrations from 30- to 60-min (mean ± 95% CI difference = −48.67 ± 10.61 mg/dL).

**Figure 4:**
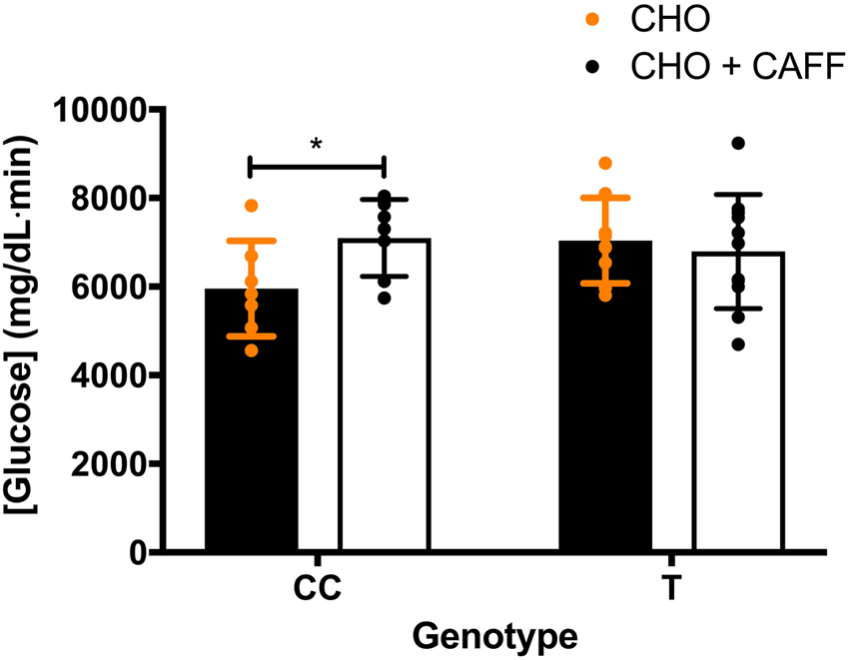
The mean ± 95% confidence interval areas under the glucose concentration curve (AUC) during the carbohydrate (CHO; closed black bars, orange circles) versus carbohydrate and caffeine (CHO + CAFF; open black bars, black circles) conditions in the ADORA2A 1976CC versus CT/TT genotypes. * indicates a significant increase from the CHO to CHO + CAFF condition for the CC genotype, p < 0.05.

A significant genotype × condition interaction (F_1,16_ = 9.254, p = 0.008, np^2^ = 0.366) was also observed for GLU AUC (Fig. 4) when examining ADORA2A 1976T > C genotype. GLU AUC was significantly higher (p = 0.01) in the 1976CC genotype during the CHO + CAFF versus the CHO condition (mean ± 95% CI difference = 1142.14 ± 664.42 mg/dL·min). However, for the CT/TT genotype, there was no difference (p = 0.64) in GLU AUC during the CHO versus CHO + CAFF conditions (mean ± 95% CI difference = 248.18 ± 711.0). GLU AUC was not significantly different between CC and CT/TT genotype in the CHO (mean ± 95% CI difference = 1085.45 ± 1034.54 mg/dL·min; p = 0.09) or CHO + CAFF (mean ± 95% CI difference = 304.87 ± 1176.13 mg/dL·min; p = 0.81) conditions.

#### CYP1A2 (−163C > A)

**Figure 5:**
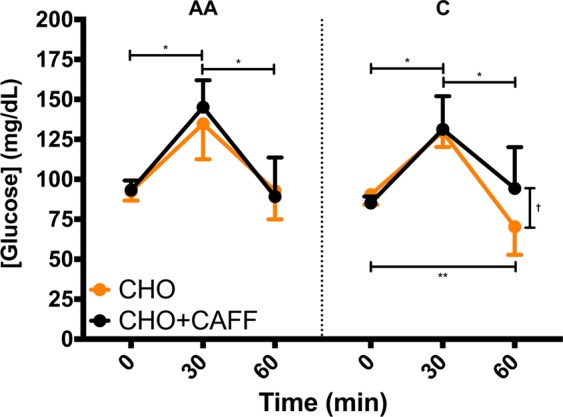
The mean ± 95% CI glucose concentrations for the CYP1A2 -163AA versus AC/CC (denoted as C) genotypes during the carbohydrate only (CHO; orange data points) versus carbohydrate and caffeine (CHO + CAFF; black data points) conditions. *Indicates difference between time points in both conditions; **Indicates difference between time points in the CHO condition only; ^†^Indicates CHO + CAFF > CHO at 60 min in AC/CC genotype, p < 0.05.

When examining CYP1A2 −163C > A genotype, there was a significant genotype × condition × time interaction (F_1,16_ = 4.534, p = 0.018, np^2^ = 0.221) for GLU responses (Fig. 5). During the CHO and CHO + CAFF conditions, there was a significant increase from BL to 30-min and a significant decrease from 30-min to 60-min in both the AA and AC/CC genotype (Fig. 5). In the AA genotype, there was no difference in [GLU] at BL versus 60-min in either the CHO (mean ± 95% CI difference = −0.27 ± 15.75 mg/dL; p ≥ 0.99) or CHO + CAFF (mean ± 95% CI difference = 4.09 ± 25.44 mg/dL; p ≥ 0.99) conditions. Similarly, in the AC/CC genotype, there was no difference (p = 0.788) in [GLU] at BL versus 60-min in the CHO + CAFF condition (mean ± 95% CI difference = −9.14 ± 24.64 mg/dL); however, [GLU] was significantly lower (p = 0.022) at 60-min than at BL during the CHO condition (mean ± 95% CI difference = 20.14 ± 13.5 mg/dL). Consequently, [GLU] was significantly greater (p = 0.029) in the AC/CC genotype during the CHO + CAFF than CHO (mean ± 95% CI difference = 24.00 ± 17.33 mg/dL) condition at 60-min, whereas there was no difference (p ≥ 0.99) in the AA genotype (mean ± 95% CI difference = −3.55 ± 21.06 mg/dL).

When examining the GLU AUC, however, there was no genotype × condition interaction (F_1,16_ = 0.010, p = 0.921, np^2^ = 0.001), or main effects for genotype (F_1,16_ = 1.108, p = 0.308, np^2^ = 0.065) or condition (F_1,16_ = 1.085, p = 0.313, np^2^ = 0.063).

## Discussion

This is the first study to examine the effects of SNPs related to caffeine metabolism on postprandial glycemic responses following acute caffeine administration and an OGTT. The results of the present study indicate that common, functional SNPs in both ADORA2A 1976T > C (rs5751876) and CYP1A2 −163C > A (rs762551) influence caffeine’s effect on postprandial glycaemia. Specifically, the ADORA2A 1976CC genotype had 14% greater [GLU] during the CHO + CAFF versus CHO condition, while the ADORA2A 1976CT/TT genotype experienced no difference between conditions (Fig. 2). In accordance with [GLU], GLU AUC was also 16% higher in the ADORA2A 1976CC genotype during the CHO + CAFF compared to the CHO condition, whereas there was no difference between conditions for the ADORA2A 1976CT/TT genotype (Fig. 4). Moreover, while the CYP1A2 −163AC/CC genotype had similar [GLU] at baseline and 30-min post-meal during the CHO and CHO + CAFF conditions, [GLU] was 16% greater at 60-min in the CHO + CAFF than CHO condition (Fig. 5). In contrast, there were no differences between conditions at baseline, 30-, or 60-min in the CYP1A2 −163AA group. Therefore, our data suggest that those with the ADORA2A 1976CC genotype experience greater post-prandial glycemic responses after consuming caffeine, whereas caffeine does not influence post-prandial glycemic responses in those with the 1976CT/TT genotype. Furthermore, those with a CYP1A2 −163AC/CC genotype have a greater post-prandial glycemic response 60-min post-meal when consuming caffeine, despite having similar glycemic responses 30-min post-meal. In contrast, those with the −163AA genotype do not appear to experience any adverse effect of caffeine on post-prandial glycemic responses.

In the present study, ADORA2A 1976CC genotype had a greater postprandial glycemic response when caffeine was consumed with a CHO meal. Interestingly, previous studies^18,22,43^ have suggested this SNP may influence physiological responses to caffeine consumption. For example, Retey *et al*.^18^ suggested that the 1976CC genotype is more likely to experience EEG patterns indicative of insomnia after caffeine consumption compared to the CT/TT genotype. On the other hand, the 1976TT genotype has been shown to be more prone to caffeine-induced anxiety^22,44^, have greater startle responses after caffeine consumption^43^, and have a higher susceptibility for panic disorder^45^. Furthermore, the 1976T > C SNP has been associated with lower coronary flow reserve in patients with cardiomyopathy^46^. Despite the apparent functional effects of the 1976T > C SNP, very few studies have provided information regarding the mechanisms by which it exerts its effects. Hohoff ^47^ observed higher availability of ADORA1 receptors in multiple regions of the brain in individuals with the 1976CT/TT genotype. Andreassi^46^ also reported greater ADORA2A protein content in peripheral blood mononuclear cells in the 1976CT/TT versus CC genotypes. Similarly, Shinohara^48^ reported higher ADORA2A protein expression in a diplotype that contained the 1976T-allele. Therefore, it is possible that the functional differences observed in CC versus CT/TT genotypes are related to differences in receptor protein content. However, this is highly speculative and future research is needed to further explore the mechanism(s) by which the 1976T > C polymorphism is influencing post-prandial glycaemia after caffeine ingestion.

We observed a 25% greater GLU response 60-min after consuming CHO + CAFF versus CHO only in the CYP1A2 −163AC/CC genotypes. In contrast, the AA genotype experienced a (non-significant) 4% greater GLU response 60-min after consuming CHO than CHO + CAFF. It has been suggested that those with the CYP1A2 −163AA genotype have higher inductility of the CYP1A2 enzyme, and are therefore thought to metabolize caffeine at a higher rate compared to the −163AC/CC genotypes^27,29^. Furthermore, it has been shown that coffee consumption is associated with increased risk of nonfatal myocardial infarction in the AC/CC genotypes only, an effect that is likely due to impaired caffeine metabolism in this population^29^. Our data appear to support the notion that individuals with the CYP1A2 −163AC/CC genotype may exhibit impaired caffeine metabolism, an effect that may adversely influence the metabolic response to a meal compared to the −163AA genotype. In fact, both the AC/CC and AA genotypes exhibited similar GLU responses at 30-min in the CHO + CAFF and CHO conditions. However, at 60-min, the addition of CAFF to the CHO meal appeared to increase the GLU response in the −163AC/CC, but not AA genotype (Fig. 5). In a recent study, Robertson *et al*.^8^ examined the influence of CYP1A2 −163C > A genotype on the metabolic response to a liquid meal tolerance test following either consumption or abstinence from coffee for a 12-week period. Following the consumption of coffee for 12 weeks, −163AA carriers exhibited higher postprandial glucose levels and suppressed fatty acid levels compared to individuals with the AC genotype, with no differences observed for serum insulin following the meal tolerance test. While the results reported by Robertson *et al*.^8^ appear to conflict with those of the present study, these dissimilarities may be due to differences in study design. Specifically, we examined the acute effect of caffeine ingestion, whereas Robertson *et al*.^8^ examined the chronic effect of caffeine consumption and did not allow participants to consume caffeine for the 12 h prior to their meal tolerance test. In addition, a glucose tolerance test was used in the present study, whereas a mixed meal tolerance test was used by Robertson *et al*.^8^. Thus, future studies are needed to further examine the interplay between acute and chronic caffeine consumption and the effects on post-prandial metabolic responses.

The present study investigated the influence of the ADORA2A 1976T > C and CYP1A2 −163C > A SNPs on the metabolic response to a carbohydrate or caffeine and carbohydrate load. It is apparent that both the 1976T > C and −163C > A SNPs significantly altered the effect that caffeine has on the GLU responses to a carbohydrate meal. Based on our data, however, we would suggest that the 1976T > C polymorphism may be more functionally relevant, because it significantly influenced the AUC. Specifically, individuals with the 1976CC genotype, who represent a little under half the population^23,35^, had greater GLU levels and a greater GLU AUC following the consumption of CHO + CAFF compared to the consumption of CHO alone. Individuals with the CYP1A2 −163AC/CC genotype, which included 39% of our sample, displayed greater GLU levels only at 60-min during the CHO + CAFF versus CHO condition, indicating that the −163C > A SNP may only influence caffeine’s effects on glucose metabolism at later time points. Overall, these results may provide further insight into the individual variability observed in the response to caffeine in both acute and longitudinal studies, and may have important implications from a public health perspective. Given that caffeine consumption has, overall, resulted in favorable outcomes in regards to health and quality of life with aging, more insight into the factors behind these health outcomes is necessary^16^. In the future, studies should directly examine caffeine and caffeine metabolite concentrations in the blood following caffeine consumption between relevant CYP and adenosine genotypes to better understand their influence on caffeine metabolism. Future studies are also needed to further explore the mechanisms by which caffeine may influence glucose responses in those with the ADORA2A 1976CC or CYP1A2 -163AC/CC genotypes.

## Supplementary information

Table S1
