# Supplementary material for: Genetic Polymorphisms in ADORA2A and CYP1A2 Influence Caffeine’s Effect on Postprandial Glycaemia
Source: Sci Rep. 2019 Jul 19;9:10532. doi: 10.1038/s41598-019-46931-0 (PMC6642114; doi:10.1038/s41598-019-46931-0)
Supplement: Supplementary file 1 — Table S1 [file 41598_2019_46931_MOESM1_ESM.pdf]

Genetic Polymorphisms in ADORA2A and CYP1A2 Influence Caffeine's Effect on Postprandial Glycaemia

Banks, N.F.[1,2], Tomko, P.M.[1,2], Colquhoun, R.J.[1,2], Muddle, T.W.D.[1,2], Emerson, S.R.[2,3], Jenkins, N.D.M.*[1,2]

[1]Applied Neuromuscular Physiology Laboratory, Oklahoma State University, Stillwater, OK [2]Laboratory for Applied Nutrition and Exercise Science, Oklahoma State University, Stillwater, OK [3]Nutritional Sciences, Oklahoma State University, Stillwater, OK

Address for Correspondence:

Nathaniel D.M. Jenkins, Ph.D.

Applied Neuromuscular Physiology Laboratory School of Kinesiology, Applied Health & Recreation Laboratory for Applied Nutrition and Exercise Science

Department of Nutritional Sciences 187 Colvin Recreation Center Oklahoma State University Stillwater, OK 74074
nathaniel.jenkins@okstate.edu

Table S1

Postprandial Glucose Response							
	CYP1A2 -163C>A	ADORA2A 1976T>C	CONDITION	GLU 0-min	GLU 30-min	GLU 60-min	GLU AUC
S01	AC	CT	CHO	92	127	90	6540
			CHO+CAFF	82	141	101	6975
S02	AA	CC	CHO	95	96	102	5835
			CHO+CAFF	98	149	73	7035
S03	AA	CT	CHO	83	127	64	6015
			CHO+CAFF	83	108	55	5310
S04	CC	CT	CHO	83	121	62	5805
			CHO+CAFF	80	88	57	4695
S05	AA	CC	CHO	75	96	71	5070
			CHO+CAFF	88	140	137	7575
S06	CC	CC	CHO	88	115	54	5580
			CHO+CAFF	89	126	67	6120
S07	AC	CC	CHO	84	134	56	6120
			CHO+CAFF	88	149	101	7305
S08	AA	TT	CHO	90	175	100	8100
			CHO+CAFF	93	124	68	6135
S10	AA	CT	CHO	104	161	113	8085
			CHO+CAFF	98	187	144	9240
S11	AA	TT	CHO	104	181	120	8790
			CHO+CAFF	97	153	78	7215
S12	AA	CC	CHO	90	144	144	7830
			CHO+CAFF	87	148	153	8040
S13	AC	CT	CHO	96	134	95	6885
			CHO+CAFF	82	149	124	7560

S14	AA	CC	CHO	93	135	83	6690
			CHO+CAFF	93	179	73	7860
S15	AC	CT	CHO	102	147	85	7215
			CHO+CAFF	92	147	131	7755
S17	AC	CT	CHO	88	130	50	5970
			CHO+CAFF	83	119	79	6000
S18	AA	TT	CHO	94	152	78	7140
			CHO+CAFF	82	134	61	6165
S19	AA	CC	CHO	90	80	54	4560
			CHO+CAFF	93	112	66	5745
S20	AA	TT	CHO	99	135	91	6900
			CHO+CAFF	114	162	73	7665
